# Supplementary material for: The international trial of nasal oxygen therapy after cardiac surgery (NOTACS) in patients at high risk of postoperative pulmonary complications: Economic evaluation protocol and analysis plan
Source: PLoS One. 2025 Jan 28;20(1):e0311861. doi: 10.1371/journal.pone.0311861 (PMC11774360; doi:10.1371/journal.pone.0311861)
Supplement: S1 Appendix — (DOCX) [file pone.0311861.s001.docx]

**S1 Appendix**


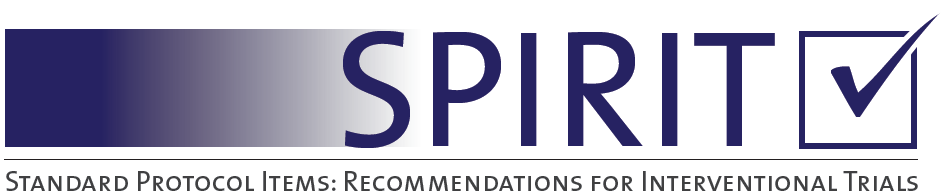
**S1 Table. SPIRIT Statement**

SPIRIT 2013 Checklist: Recommended items to address in a clinical trial protocol and related documents*

| Section/item | Item No | Description | Addressed on page number |
| --- | --- | --- | --- |
| **Administrative information** | | |  |
| Title | 1 | Descriptive title identifying the study design, population, interventions, and, if applicable, trial acronym | Page 1, lines 3 to 5 |
| Trial registration | 2a | Trial identifier and registry name. If not yet registered, name of intended registry | Page 3, lines 52 to 54 |
|  | 2b | All items from the World Health Organization Trial Registration Data Set | Page 3, lines 52 to 54 |
| Protocol version | 3 | Date and version identifier | Page 5, lines 103 to 104 |
| Funding | 4 | Sources and types of financial, material, and other support | Submission system |
| Roles and responsibilities | 5a | Names, affiliations, and roles of protocol contributors | Page 1, line 10 to page 2, line 24 |
|  | 5b | Name and contact information for the trial sponsor | Please refer to section {5b} Table in the SPIRIT format published main trial protocol available at  [**https://rdcu.be/dU3Ec**](https://rdcu.be/dU3Ec) |
|  | 5c | Role of study sponsor and funders, if any, in study design; collection, management, analysis, and interpretation of data; writing of the report; and the decision to submit the report for publication, including whether they will have ultimate authority over any of these activities | Please refer to section {5c} Table in the SPIRIT format published main trial protocol available at  [**https://rdcu.be/dU3Ec**](https://rdcu.be/dU3Ec) |
| Introduction |  |  |  |
| Background and rationale | 6a | Description of research question and justification for undertaking the trial, including summary of relevant studies (published and unpublished) examining benefits and harms for each intervention | Page 4, lines 72 to 79, Page 5, lines 88 to 104 |
|  | 6b | Explanation for choice of comparators | Please refer to section {6b} in the SPIRIT format published main trial protocol available at  [**https://rdcu.be/dU3Ec**](https://rdcu.be/dU3Ec) |
| Objectives | 7 | Specific objectives or hypotheses | Page 5, lines 97 to 104 |
| Trial design | 8 | Description of trial design including type of trial (eg, parallel group, crossover, factorial, single group), allocation ratio, and framework (eg, superiority, equivalence, noninferiority, exploratory) | Page 6, lines 108 to 120 |
| Methods: Participants, interventions, and outcomes | | |  |
| Study setting | 9 | Description of study settings (eg, community clinic, academic hospital) and list of countries where data will be collected. Reference to where list of study sites can be obtained | Page 6, lines 108 to 120 |
| Eligibility criteria | 10 | Inclusion and exclusion criteria for participants. If applicable, eligibility criteria for study centres and individuals who will perform the interventions (eg, surgeons, psychotherapists) | Please refer to section {10} in the SPIRIT format published main trial protocol available at  [**https://rdcu.be/dU3Ec**](https://rdcu.be/dU3Ec) |
| Interventions | 11a | Interventions for each group with sufficient detail to allow replication, including how and when they will be administered | Page 6, line 121 to page 7, line 134 |
|  | 11b | Criteria for discontinuing or modifying allocated interventions for a given trial participant (eg, drug dose change in response to harms, participant request, or improving/worsening disease) | Page 6, line 121 to page 7, line 134 |
|  | 11c | Strategies to improve adherence to intervention protocols, and any procedures for monitoring adherence (eg, drug tablet return, laboratory tests) | Please refer to section {11c} in the SPIRIT format published main trial protocol available at  [**https://rdcu.be/dU3Ec**](https://rdcu.be/dU3Ec) |
|  | 11d | Relevant concomitant care and interventions that are permitted or prohibited during the trial | Please refer to section {11d} in the SPIRIT format published main trial protocol available at  [**https://rdcu.be/dU3Ec**](https://rdcu.be/dU3Ec) |
| Outcomes | 12 | Primary, secondary, and other outcomes, including the specific measurement variable (eg, systolic blood pressure), analysis metric (eg, change from baseline, final value, time to event), method of aggregation (eg, median, proportion), and time point for each outcome. Explanation of the clinical relevance of chosen efficacy and harm outcomes is strongly recommended | Page 15, line 227 to Page 18, line 288.  Page 20, line 346 to Page 21, line 368 |
| Participant timeline | 13 | Time schedule of enrolment, interventions (including any run-ins and washouts), assessments, and visits for participants. A schematic diagram is highly recommended (see Figure) | Figure 1  Page 7, line 146 to Page 8, line 156 |
| Sample size | 14 | Estimated number of participants needed to achieve study objectives and how it was determined, including clinical and statistical assumptions supporting any sample size calculations | Page 6, lines 110 to 113.  Please also refer to section {14} in the SPIRIT format published main trial protocol available at  [**https://rdcu.be/dU3Ec**](https://rdcu.be/dU3Ec) |
| Recruitment | 15 | Strategies for achieving adequate participant enrolment to reach target sample size | Please refer to section {15} in the SPIRIT format published main trial protocol available at  [**https://rdcu.be/dU3Ec**](https://rdcu.be/dU3Ec) |
| **Methods: Assignment of interventions (for controlled trials)** | | |  |
| Allocation: |  |  |  |
| Sequence generation | 16a | Method of generating the allocation sequence (eg, computer-generated random numbers), and list of any factors for stratification. To reduce predictability of a random sequence, details of any planned restriction (eg, blocking) should be provided in a separate document that is unavailable to those who enrol participants or assign interventions | Please refer to section {16a} in the SPIRIT format published main trial protocol available at  [**https://rdcu.be/dU3Ec**](https://rdcu.be/dU3Ec) |
| Allocation concealment mechanism | 16b | Mechanism of implementing the allocation sequence (eg, central telephone; sequentially numbered, opaque, sealed envelopes), describing any steps to conceal the sequence until interventions are assigned | Please refer to section {16b} in the SPIRIT format published main trial protocol available at  [**https://rdcu.be/dU3Ec**](https://rdcu.be/dU3Ec) |
| Implementation | 16c | Who will generate the allocation sequence, who will enrol participants, and who will assign participants to interventions | Please refer to section {16c} in the SPIRIT format published main trial protocol available at  [**https://rdcu.be/dU3Ec**](https://rdcu.be/dU3Ec) |
| Blinding (masking) | 17a | Who will be blinded after assignment to interventions (eg, trial participants, care providers, outcome assessors, data analysts), and how | Please refer to section {17a} in the SPIRIT format published main trial protocol available at  [**https://rdcu.be/dU3Ec**](https://rdcu.be/dU3Ec) |
|  | 17b | If blinded, circumstances under which unblinding is permissible, and procedure for revealing a participant’s allocated intervention during the trial | Please refer to section {17b} in the SPIRIT format published main trial protocol available at  [**https://rdcu.be/dU3Ec**](https://rdcu.be/dU3Ec) |
| **Methods: Data collection, management, and analysis** | | |  |
| Data collection methods | 18a | Plans for assessment and collection of outcome, baseline, and other trial data, including any related processes to promote data quality (eg, duplicate measurements, training of assessors) and a description of study instruments (eg, questionnaires, laboratory tests) along with their reliability and validity, if known. Reference to where data collection forms can be found, if not in the protocol | Please refer to section {18a} in the SPIRIT format published main trial protocol available at  [**https://rdcu.be/dU3Ec**](https://rdcu.be/dU3Ec) |
|  | 18b | Plans to promote participant retention and complete follow-up, including list of any outcome data to be collected for participants who discontinue or deviate from intervention protocols | Please refer to section {18b} in the SPIRIT format published main trial protocol available at  [**https://rdcu.be/dU3Ec**](https://rdcu.be/dU3Ec) |
| Data management | 19 | Plans for data entry, coding, security, and storage, including any related processes to promote data quality (eg, double data entry; range checks for data values). Reference to where details of data management procedures can be found, if not in the protocol | Page 18, lines 293 to 299  Please also refer to section {19} in the SPIRIT format published main trial protocol available at  [**https://rdcu.be/dU3Ec**](https://rdcu.be/dU3Ec) |
| Statistical methods | 20a | Statistical methods for analysing primary and secondary outcomes. Reference to where other details of the statistical analysis plan can be found, if not in the protocol | Page 19, line 322 to Page 21, line 368  Please also refer to the statistical analysis protocol at [**https://rdcu.be/dU3MZ**](https://rdcu.be/dU3MZ) |
|  | 20b | Methods for any additional analyses (eg, subgroup and adjusted analyses) | Page 24, line 375 to page 25, line 401 |
|  | 20c | Definition of analysis population relating to protocol non-adherence (eg, as randomised analysis), and any statistical methods to handle missing data (eg, multiple imputation) | Page 18, line 292 to page 19, line 320 |
| **Methods: Monitoring** | | |  |
| Data monitoring | 21a | Composition of data monitoring committee (DMC); summary of its role and reporting structure; statement of whether it is independent from the sponsor and competing interests; and reference to where further details about its charter can be found, if not in the protocol. Alternatively, an explanation of why a DMC is not needed | Please refer to section {21a} in the SPIRIT format published main trial protocol available at  [**https://rdcu.be/dU3Ec**](https://rdcu.be/dU3Ec) |
|  | 21b | Description of any interim analyses and stopping guidelines, including who will have access to these interim results and make the final decision to terminate the trial | Please refer to section {21b} in the SPIRIT format published main trial protocol available at  [**https://rdcu.be/dU3Ec**](https://rdcu.be/dU3Ec) |
| Harms | 22 | Plans for collecting, assessing, reporting, and managing solicited and spontaneously reported adverse events and other unintended effects of trial interventions or trial conduct | Please refer to section {22} in the SPIRIT format published main trial protocol available at  [**https://rdcu.be/dU3Ec**](https://rdcu.be/dU3Ec) |
| Auditing | 23 | Frequency and procedures for auditing trial conduct, if any, and whether the process will be independent from investigators and the sponsor | Please refer to section {23} in the SPIRIT format published main trial protocol available at  [**https://rdcu.be/dU3Ec**](https://rdcu.be/dU3Ec) |
| Ethics and dissemination | | |  |
| Research ethics approval | 24 | Plans for seeking research ethics committee/institutional review board (REC/IRB) approval | Page 27, lines 443 to 453  Submission system |
| Protocol amendments | 25 | Plans for communicating important protocol modifications (eg, changes to eligibility criteria, outcomes, analyses) to relevant parties (eg, investigators, REC/IRBs, trial participants, trial registries, journals, regulators) | Please refer to section {25} in the SPIRIT format published main trial protocol available at  [**https://rdcu.be/dU3Ec**](https://rdcu.be/dU3Ec) |
| Consent or assent | 26a | Who will obtain informed consent or assent from potential trial participants or authorised surrogates, and how (see Item 32) | Please refer to section {26a} in the SPIRIT format published main trial protocol available at  [**https://rdcu.be/dU3Ec**](https://rdcu.be/dU3Ec) |
|  | 26b | Additional consent provisions for collection and use of participant data and biological specimens in ancillary studies, if applicable | Please refer to section {26b} in the SPIRIT format published main trial protocol available at  [**https://rdcu.be/dU3Ec**](https://rdcu.be/dU3Ec) |
| Confidentiality | 27 | How personal information about potential and enrolled participants will be collected, shared, and maintained in order to protect confidentiality before, during, and after the trial | Please refer to section {27} in the SPIRIT format published main trial protocol available at  [**https://rdcu.be/dU3Ec**](https://rdcu.be/dU3Ec) |
| Declaration of interests | 28 | Financial and other competing interests for principal investigators for the overall trial and each study site | Submission system |
| Access to data | 29 | Statement of who will have access to the final trial dataset, and disclosure of contractual agreements that limit such access for investigators | Please refer to section {29} in the SPIRIT format published main trial protocol available at  [**https://rdcu.be/dU3Ec**](https://rdcu.be/dU3Ec) |
| Ancillary and post-trial care | 30 | Provisions, if any, for ancillary and post-trial care, and for compensation to those who suffer harm from trial participation | Please refer to section {30} in the SPIRIT format published main trial protocol available at  [**https://rdcu.be/dU3Ec**](https://rdcu.be/dU3Ec) |
| Dissemination policy | 31a | Plans for investigators and sponsor to communicate trial results to participants, healthcare professionals, the public, and other relevant groups (eg, via publication, reporting in results databases, or other data sharing arrangements), including any publication restrictions | Page 27, lines 443 to 453 |
|  | 31b | Authorship eligibility guidelines and any intended use of professional writers | Submission system |
|  | 31c | Plans, if any, for granting public access to the full protocol, participant-level dataset, and statistical code | Please refer to section {31c} in the SPIRIT format published main trial protocol available at  [**https://rdcu.be/dU3Ec**](https://rdcu.be/dU3Ec) |
| Appendices |  |  |  |
| Informed consent materials | 32 | Model consent form and other related documentation given to participants and authorised surrogates | Not applicable |
| Biological specimens | 33 | Plans for collection, laboratory evaluation, and storage of biological specimens for genetic or molecular analysis in the current trial and for future use in ancillary studies, if applicable | Not applicable |

*It is strongly recommended that this checklist be read in conjunction with the SPIRIT 2013 Explanation & Elaboration for important clarification on the items. Amendments to the protocol should be tracked and dated. The SPIRIT checklist is copyrighted by the SPIRIT Group under the Creative Commons “[Attribution-NonCommercial-NoDerivs 3.0 Unported](http://www.creativecommons.org/licenses/by-nc-nd/3.0/)” license.

Source: <https://www.acpjournals.org/doi/10.7326/0003-4819-158-3-201302050-0058>

Citation: An-Wen Chan, Jennifer M. Tetzlaff, Douglas G. Altman, et al. SPIRIT 2013 Statement: Defining Standard Protocol Items for Clinical Trials. Ann Intern Med.2013; 158:200-207. [Epub 5 February 2013]. doi:10.7326/0003-4819-158-3-201302050-00583

The referenced articles in the SPIRIT STATEMENT checklist refer to the main trial protocol (Trial registration ID: ISRCTN14092678) and the statistical analysis plans available publicly on the following links:

NOTACS main trial protocol paper (published as per SPIRIT statement): <https://rdcu.be/dU3Ec>

Citation: Earwaker, M., Villar, S., Fox-Rushby, J. et al. Effect of high-flow nasal therapy on patient-centred outcomes in patients at high risk of postoperative pulmonary complications after cardiac surgery: a study protocol for a multicentre adaptive randomised controlled trial. Trials 23, 232 (2022). <https://doi.org/10.1186/s13063-022-06180-5>

NOTACS statistical analysis plan: <https://rdcu.be/dU3MZ>

Citation: Dawson, S.N., Chiu, YD., Klein, A.A. et al. Effect of high-flow nasal therapy on patient-centred outcomes in patients at high risk of postoperative pulmonary complications after cardiac surgery: a statistical analysis plan for NOTACS, a multicentre adaptive randomised controlled trial. Trials 23, 699 (2022). <https://doi.org/10.1186/s13063-022-06607-z>

**S2 Table. Summary of changes made to data collection tools in Australia and New Zealand compared with United Kingdom**

| **Country** | **Data collection** | **Description of changes** |
| --- | --- | --- |
| **Resource utilization** | | |
| Australia | Health and personal social service resource use – baseline | Includes country specific patient details on ethnicity, planned surgical intervention options and a separate section for Euro SCORE II. |
|  | Health and personal social service resource use – procedure | Includes additional reporting of post-operative bloods (haematocrit, CRP, Platelets, white cell count). |
|  | Health and personal social service resource use – patient family resource utilisation | Includes additional option of speaking to a nurse or doctor by telephone, telehealth or online (compared with only telephone in UK) |
|  | Patient costs – at baseline | Includes country specific questions on education and household income and an additional question on healthcare concession care. |
|  | Patient costs – at discharge | Includes additional option for out-of-pocket payment amount for ambulance travel |
|  | Patient costs – at follow-up | Includes additional out-of-pocket payment amount for ambulance travel, health professional visits and telephonic consultation. Excludes NHS staff telephone consultations. |
| New Zealand | Health and personal social service resource use – baseline | Includes country specific patient details on ethnicity, planned surgical intervention options and a separate section for Euro SCORE II. |
|  | Patient costs – at baseline | Includes country specific questions on education and household income (weekly/yearly). |
|  | Patient costs – at discharge | Includes additional option for out-of-pocket payment amount for ambulance travel |
|  | Patient costs – at follow-up | Includes additional out-of-pocket payment amount for ambulance travel, health professional visits and telephonic consultation. Excludes NHS staff telephone consultations. |
| **Health outcomes** | | |
| Australia | EQ-5D-5L | Uses EuroQol approved EQ-5D-5L questionnaire for Australia and Australian value set developed by Norman et al. |
| New Zealand | EQ-5D-5L | Uses EuroQol approved EQ-5D-5L questionnaire for New Zealand and New Zealand value set developed by Sullivan et al. |

**Patient and public involvement in data collection:**

The patient and public involvement (PPI) group in the UK guided the trial design and data collection methods. First, the primary outcome for the trial, days at home (DAH), the most important outcome measure in the short term, was selected by the PPI group during the study design and application process. The most appropriate time of approach and consent has been informed from the feedback from the PPI group. The identification of costs falling on patients was initially based on a search for validated patient cost questionnaires located in the database of instruments for resource use measurement (DIRUM), and publications that had measured costs falling on patients in this intervention area. As neither search was productive, one patient and their main home-based carer who had experienced HFNT in the UK were interviewed both before and after developing the patient cost questionnaire. The first, online, interview lasted 45 minutes and focussed on the largest and most frequent time and money costs they experienced when in hospital and 3 months after leaving the hospital. Following the interview, questionnaires in the DIRUM were considered, but none were selected as no questionnaire matched well enough. The second interview was face to face, lasted for an hour, and focussed on their views of how to improve the questionnaire, which both patient and carer had received and reviewed in advance of the interview. The questionnaire was then reviewed by the trial steering committee.

**S3 Table. Amendments to data collection methods**

| **Data collection for intravenous (IV) medications recorded during index admission for surgery** | |
| --- | --- |
| Issue | **Initial Data Collection Method**: Recorded all titration changes for each medication administered during inpatient admission.  **Problem**: Resource-intensive, increased the data collection burden on the research staff. |
| Amendment | A list of most prescribed IV medications was developed using already collected data. Data collection staff were trained to select all patient specific prescribed IV medications at the time of discharge from the list of most common IV medications and to add any new drugs that were not listed. |
| Amendment implementation | The amendment was implemented in the United Kingdom, Australia and New Zealand on 23/08/2022. |
| Proposed method of analysis post-amendment | The average dosage and strength of consumption of a particular drug observed in the initially collected cases will be used to apportion the overall consumption of IV medications during the period of stay for patients recruited post-amendment. For any new IV medication not listed, relevant literature will be used to allocate the number of units utilized. |
| **Data collection for medications from discharge following index admission to 90-days i.e. end of follow-up** | |
| Issue | **Initial Data Collection Method**: Patients expected to record medication data consistently throughout follow-up period.  **Problem**: Data collection burden on patients led to reduced patient compliance, resulting in a poor return rate of the participant location and medication diary. |
| Amendment | The participant location and medication diary were simplified to record medications at three-time points; at discharge, on day 30 after surgery, and day 90 after surgery. Data collection at these points now identify all medications consumed with dosage, frequency, route of administration, and number of days the patient was advised to take the medication |
| Amendment implementation | Amendment was approved (UK research ethics committee - Substantial Amendment 3 – 12/12/2022) and implemented in the United Kingdom on 25/04/2023 and in Australia and New Zealand on 12/05/2023. |
| Proposed method of analysis post-amendment | Medication data on dosage, frequency, route of administration, and number of days consumption collected on the day of discharge, on day 30 after surgery, and day 90 after surgery will be used to quantify medication consumption over 90 days. For life-long medications, 90 days of use will be assumed. For patients not recalling or reporting the number of days of medication use, an assumption will be made depending on the nature of the medication itself. For example: For a given antibiotic, an average expected course duration as prescribed by British National Formulary (BNF) along with Prescription Cost Analysis (PCA) for costs will be used for UK patients. |
