# Supplementary material for: The international trial of nasal oxygen therapy after cardiac surgery (NOTACS) in patients at high risk of postoperative pulmonary complications: Economic evaluation protocol and analysis plan
Source: PLoS One. 2025 Jan 28;20(1):e0311861. doi: 10.1371/journal.pone.0311861 (PMC11774360; doi:10.1371/journal.pone.0311861)
Supplement: S2 Appendix — (DOCX) [file pone.0311861.s002.docx]

**S2 Appendix**

Table S1. List of NOTACS investigators

| **Name** | **Role** | **Affiliation** |
| --- | --- | --- |
| **UK** | | |
| Dr. Andrew Klein  (Lead author)  Email ID:  [andrew.klein@nhs.net](mailto:andrew.klein@nhs.net) | Chief Investigator; Consultant Anaesthetist | Department of Anaesthesia, Royal Papworth Hospital |
| Jo Steele | Senior Data Manager | Papworth Trials Unit Collaboration, Royal Papworth Hospital |
| Ayman Mohammed | Data Manager | Papworth Trials Unit Collaboration, Royal Papworth Hospital |
| Ellen Temple | Clinical Trial Manager | Papworth Trials Unit Collaboration, Royal Papworth Hospital |
| Fiona Bottrill | Study Monitor; QA Project Manager | Papworth Trials Unit Collaboration, Royal Papworth Hospital |
| Dr. Sofia S. Villar | Senior Statistician; MRC Investigator - Programme Leader | MRC Biostatistics Unit, University of Cambridge |
| Prof Gudrun Kunst | Consultant Anaesthetist & Professor of Cardiovascular Anaesthesia | Department of Anaesthetics and Pain Therapy, King’s College Hospital NHS FT, London; School of Cardiovascular and Metabolic Medicine & Sciences, King’s College London |
| Prof Gavin J Murphy | Academic Cardiac Surgeon; Director of Leicester Clinical Trials Unit | Leicester NIHR Biomedical Research Centre |
| Dr. Guillermo Martinez | Principal Investigator; Consultant Anaesthetist | Department of Anaesthesia, Royal Papworth Hospital NHS Foundation Trust |
| Carol Freeman | Clinical Project Manager (former) | Papworth Trials Unit Collaboration, Royal Papworth Hospital |
| Melissa Earwaker | Trial Manager (former) | Papworth Trials Unit Collaboration, Royal Papworth Hospital |
| Yi-Da Chiu | Study Statistician (former) | Papworth Trials Unit Collaboration, Royal Papworth Hospital |
| Dr. Vasileios Zochios | Consultant in Adult ECMO and Intensive Care Medicine; Honorary Senior Lecturer | University Hospitals of Leicester NHS Trust, Glenfield Hospital ECMO Unit, Glenfield, Leicester, United Kingdom;  Department of Cardiovascular Sciences, University of Leicester, Leicester, United Kingdom |
| Val Brown | PPI Representative (former, withdrew) | NA |
| Robin McClean | PPI Representative (former, withdrew) | NA |
| Geoff Brown | PPI Representative (former, deceased) | NA |
| **Australia** | | |
| A/Prof. Edward Litton | Chief Investigator Australia; Head of ICU Research | Curtin School of Population Health, Curtin University, Fiona Stanley Hospital, Perth Western Australia |
| Dr. Julieann Coombes | Australian Management Co-committee Chair; Program Lead, Indigenous Methodologies and Systems Change, Guunu-maana (Heal) Aboriginal & Torres Strait Islander Health | The George Institute for Global Health, Australia |
| Camila Kairuz Santos | Australian sub-study support | The George Institute for Global Health, Australia |
| Prof David Pilcher | Principal Investigator; Senior Intensive Care Specialist; Adjunct Clinical Professor | Department of Intensive Care, The Alfred Hospital, Melbourne; The Australian and New Zealand Intensive Care – Research Centre, Monash University, Melbourne, Victoria |
| Prof Andrew Maiorana | Professor of Clinical Exercise Physiology; Head of the Department of Exercise Physiology | Curtin School of Allied Health, Curtin University; Fiona Stanley Hospital, Perth, Western Australia |
| Prof Christopher Reid | John Curtin Distinguished Professor; Cardiovascular Epidemiologist | School of Public Health and Preventive Medicine, Monash University; Curtin School of Population Health, Curtin University, Perth, Western Australia |
| Dr Sumit Yadav | Specialist Cardiothoracic Surgeon; Senior Lecturer | Mater Private Hospital Townsville; James Cook University, Townsville, Queensland |
| Dr Mahesh Ramanan | Principal Investigator; Staff Specialist in Intensive Care Medicine | The Prince Charles Hospital, Brisbane, Australia, Brisbane, Queensland |
| Dr Siva Senthuran | Principal Investigator; Senior Specialist in Intensive Care Medicine | Townsville University Hospital, Australia, Townsville, Queensland |
| Keziah Bennett-Brook | Program Head of the Guunu-maana (Heal) Aboriginal and Torres Strait Islander Health Program | The George Institute for Global Health, Australia, NSW |
| A/Prof Anthony Delaney | Principal Investigator; Senior Staff Specialist; Associate Professor | Malcolm Fisher Department of Intensive Care Medicine, Royal North Shore Hospital; Faculty of Medicine and Health, The University of Sydney, NSW |
| Prof Neil Orford | Principal Investigator; Senior Staff Specialist in Intensive Care; Associate Professor of Intensive Care Medicine | University Hospital Geelong; Deakin University School of Medicine; Geelong, Victoria |
| **New Zealand** | | |
| A/Prof Rachael Parke | Principal Investigator, Associate Professor; Nurse Senior Research Fellow | School of Nursing, University of Auckland; Cardiothoracic and Vascular ICU at Auckland City Hospital, Auckland, New Zealand |
| Dr Shay McGuinness | Principal Investigator; Intensive Care Specialist | Cardiothoracic and Vascular ICU at Auckland City Hospital, Auckland, New Zealand |

Table S2. List of NOTACS Trial Steering Committee members

| **Name** | **Role** | **Affiliation** |
| --- | --- | --- |
| Prof Stephen Brett  (Lead author)  Email ID:  [stephen.brett@imperial.ac.uk](mailto:stephen.brett@imperial.ac.uk) | Independent Chair; Professor of Critical Care; Consultant in Intensive Care Medicine | Dept of Surgery and Cancer, Imperial College London; Imperial College Healthcare NHS Trust |
| Prof Susan Griffin | Independent Health Economist; Professor of Health Economics | Centre for Health Economics, University of York |
| Dr Philip Pallmann | Independent Statistician; Principal Research Fellow (Statistics) | Centre for Trials Research,  College of Biomedical and Life Sciences,  Cardiff University |
| Dr Peter Shirley | Independent Clinician; Consultant in Intensive Care Medicine and Anaesthesia | Royal London Hospital, Barts Health NHS Trust |
| Andrew Hoppington | PPI Representative | N/A |
| Dr. Marijcke Veltman | PPI Representative | N/A |

Table S3. List of NOTACS Data Monitoring and Ethics Committee (DMEC) members

| **Name** | **Role** | **Affiliation** |
| --- | --- | --- |
| Prof Mahmoud Loubani  (Lead author)  Email ID: [mahmoud.loubani@nhs.net](mailto:mahmoud.loubani@nhs.net) | Independent Chair | Hull University Teaching Hospitals NHS Trust |
| Prof Graeme MacLennan | Independent Statistician | University of Aberdeen |
| Dr Peter Alston | Independent Cardiac Anaesthetist | Royal Infirmary of Edinburgh |
| Prof Thomas Jaki | Independent Statistician  (former) | Lancaster University |
