# Supplementary material for: The international trial of nasal oxygen therapy after cardiac surgery (NOTACS) in patients at high risk of postoperative pulmonary complications: Economic evaluation protocol and analysis plan
Source: PLoS One. 2025 Jan 28;20(1):e0311861. doi: 10.1371/journal.pone.0311861 (PMC11774360; doi:10.1371/journal.pone.0311861)
Supplement: Attachment — Submitted filename: Response to reviewers_final.docx [file pone.0311861.s004.docx]

**Authors’ Responses to Peer Review**

Manuscript Number: PONE-D-24-41068

Manuscript Title: The international trial of nasal oxygen therapy after cardiac surgery (NOTACS) in patients at high risk of postoperative pulmonary complications: Economic evaluation protocol and analysis plan

| **Editor’s Comments** |  |
| --- | --- |
| Formatting:  1.  Please ensure that your manuscript meets PLOS ONE's style requirements, including those for file naming. The PLOS ONE style templates can be found at | We sincerely thank you for considering the manuscript and allowing us to further improve the manuscript based on the valuable feedback we have received from the reviewers.  We have reviewed the manuscript against formatting guidelines to ensure that manuscript meets PLOS ONE’s style requirements to the best of our knowledge. |
| 2. When completing the data availability statement of the submission form, you indicated that you will make your data available on acceptance. We strongly recommend all authors decide on a data sharing plan before acceptance, as the process can be lengthy and hold up publication timelines. Please note that, though access restrictions are acceptable now, your entire data will need to be made freely accessible if your manuscript is accepted for publication. This policy applies to all data except where public deposition would breach compliance with the protocol approved by your research ethics board. If you are unable to adhere to our open data policy, please kindly revise your statement to explain your reasoning and we will seek the editor's input on an exemption. Please be assured that, once you have provided your new statement, the assessment of your exemption will not hold up the peer review process. | As this an economic evaluation protocol study, the study currently does not have any datasets generated or analysed. We do not expect to have any datasets generated from this current protocol paper. In accordance with the data management plan for the final study completion, we request the data availability statement to be revised as follows:  “The data that support the findings of this study will be made available following journal and funder requirements. Details of data accessibility will be included when the results of the study are published.” |
| 3. PLOS requires an ORCID iD for the corresponding author in Editorial Manager on papers submitted after December 6th, 2016. Please ensure that you have an ORCID iD and that it is validated in Editorial Manager. To do this, go to ‘Update my Information’ (in the upper left-hand corner of the main menu), and click on the Fetch/Validate link next to the ORCID field. This will take you to the ORCID site and allow you to create a new iD or authenticate a pre-existing iD in Editorial Manager. | Apologies, the ORCID ID for the corresponding author has now been updated on the PLOS ONE submission system. |
| 4. One of the noted authors is a group or consortium [NOTACS investigators, NOTACS Trial Steering Committee and NOTACS DMEC Committee]. In addition to naming the author group, please list the individual authors and affiliations within this group in the acknowledgments section of your manuscript. Please also indicate clearly a lead author for this group along with a contact email address. | The PLOS ONE submission guidelines suggest ‘You may include the full list of members in the Acknowledgments or in a supporting information file.’  As we have a long list of authors across the three groups, we have chosen to report these author names and their affiliations in supporting information file (File S2 Appendix). The revised manuscript now indicates lead author for the group with contact email address. |
| 5. Your ethics statement should only appear in the Methods section of your manuscript. If your ethics statement is written in any section besides the Methods, please move it to the Methods section and delete it from any other section. Please ensure that your ethics statement is included in your manuscript, as the ethics statement entered into the online submission form will not be published alongside your manuscript. | The ethics statement is now included in the methods section on page number 06, lines 108 to 117 in the revised manuscript. |
| 6. We note that there is identifying data in the Supporting Information file <S2 Appendix PLOS ONE_DMEC.docx>. Due to the inclusion of these potentially identifying data, we have removed this file from your file inventory. Prior to sharing human research participant data, authors should consult with an ethics committee to ensure data are shared in accordance with participant consent and all applicable local laws.  Data sharing should never compromise participant privacy. It is therefore not appropriate to publicly share personally identifiable data on human research participants. The following are examples of data that should not be shared:  -Name, initials, physical address  -Ages more specific than whole numbers  -Internet protocol (IP) address  -Specific dates (birth dates, death dates, examination dates, etc.)  -Contact information such as phone number or email address  -Location data  -ID numbers that seem specific (long numbers, include initials, titled “Hospital ID”) rather than random (small numbers in numerical order)  Data that are not directly identifying may also be inappropriate to share, as in combination they can become identifying. For example, data collected from a small group of participants, vulnerable populations, or private groups should not be shared if they involve indirect identifiers (such as sex, ethnicity, location, etc.) that may risk the identification of study participants.  Additional guidance on preparing raw data for publication can be found in our Data Policy.  Please remove or anonymize all personal information, ensure that the data shared are in accordance with participant consent, and re-upload a fully anonymized data set. Please note that spreadsheet columns with personal information must be removed and not hidden as all hidden columns will appear in the published file. | The S2 Appendix provides list of authors that form part of group authorship for this article. This has been framed as per PLOS ONE guidance to include the full list of members either in the acknowledgments or in a supporting information file. As we have a long list of authors within three groups, we have chosen to report these names and their affiliation as a supporting file.  We have now removed the list of participating sites as a precaution to prevent any compromise with regards to participant privacy. |
| 7. We note that the original protocol that you have uploaded as a Supporting Information file contains an institutional logo. As this logo is likely copyrighted, we ask that you please remove it from this file and upload an updated version upon resubmission. | Apologies. The revised file submission for original protocol now has all institutional logos removed. |
| 8. Please review your reference list to ensure that it is complete and correct. If you have cited papers that have been retracted, please include the rationale for doing so in the manuscript text, or remove these references and replace them with relevant current references. Any changes to the reference list should be mentioned in the rebuttal letter that accompanies your revised manuscript. If you need to cite a retracted article, indicate the article’s retracted status in the References list and also include a citation and full reference for the retraction notice. | To our knowledge, we have ensured that the reference list is complete and correct. We have not included any references to articles that have been retracted. |
| **Reviewer 1 comments** |  |
| The study addresses an interesting topic. The description of each phase is clear and detailed. I have a few remarks on the data analysis only. In details: | We sincerely thank you for reviewing and providing your valuable feedback to improve this manuscript. We are pleased to know that you think the manuscript reads well.  Following your comments, we have further revised the manuscript, and these can be reviewed as track changes. |
| 1. The definition of an outlier is always with respect to a specific distribution. A value that might be atypical with respect to, e.g., the Poisson distribution may be not atypical for the Geometric, the Negative Binomial or the Conway Maxwell Poisson distributions, just to mention the most widely used count data distribution (along with their zero-inflated counterparts). Similarly, I am strongly against the transformation of the data. It is not the data that should adapt to a specific model, but rather it is important to find the most appropriate model for the data at hand; log-transforming the data leads to a different shape and scale. | We agree that we should consider outliers in relation to the specific distribution underlying the data. We agree and will focus on understanding the data characteristics to guide our choice of distribution and the resulting model to fit the available data. We have now revised the manuscript on page 19, lines 312 to 313 to state the following:  “We will examine the data characteristics to select candidate distribution and assess model fit using information criteria, to ensure the best representation of the data.” |
| 2. Similarly, I do not really get how you can say that a missing value is MAR, MCAR or MNAR. Sensitivity analysis is often considered, under different missing data mechanisms (please refer to <https://www.routledge.com/Handbook-of-Missing-Data-Methodology/Molenberghs-Fitzmaurice-Kenward-Tsiatis-Verbeke/p/book/9780367739294> for further details). Thus, I strongly suggest to discuss more details on the way missing data will be treated, allowing for NMAR mechanisms. The authors state that multiple imputation is used, but it is not clear how many datasets will be considered and how the imputed datasets will be checked, etc. | We have revised the manuscript to dedicate more to our investigation of mechanisms of missingness. We now report the use of logistic regression to rule out MCAR mechanism and an assumption of MAR mechanism otherwise. We report that sensitivity analysis will assess the impact of using a MNAR mechanism using pattern mixture models.  The revised manuscript also now reports that the number of imputed datasets will be higher than the percentage of patients with missing data in the subset of variables included in the missing data mechanism tests and that validation of imputation will be carried about by comparison distribution of imputed data to observed values.  The revised manuscript reflects these changes on Page 19 to 20, lines 315 to 339 that reads as follows:  “The pattern and amount of missing data at each time point will be summarised using frequency and percentage plots, with pattern of missingness for resource use and outcomes. We will use logistic regression to investigate if missingness can be predicted by baseline or observed outcomes at each data collection point (54). If none of the missing variables are predicted by other variables, a missing completely at random (MCAR) mechanism will be assumed and if the percentage of missing data is below 5% for resource use and outcomes of the primary analysis, a complete case analysis (CCA) will be undertaken. This is unlikely to be the case due to the need to aggregate resource use and costs across many variables. In this case, a missing at random (MAR) mechanism will be assumed and multiple imputation (MI) methods will be used. Sensitivity analysis will assess the impact of using a missing not at random (MNAR) mechanism using pattern mixture model approach (55,56). We will use multiple imputation chained equations (MICE) with predictive mean matching, stratified by treatment arm and country to estimate missing cost and outcome data for base-case. We will explore censoring as a special case of missing data, particularly right censoring from loss to follow-up to judge the impact on estimating mean values for costs, DAH90 and QALYs using an inverse probability weighting approach (54). Imputation models will be determined during analysis as it is important that the model includes variables that are good predictors of missing variables. We will consider variables such as age, sex, baseline Barthel and/or EQ-5D-5L scores, baseline health service use, BMI, ethnicity, number of co-morbidities, whether surgery is elective, Euro Score II, ASA, and ARISCAT score. The number of imputed data sets will be higher than the percentage of patients with missing data in the subset of variables included in the missing data mechanism tests to achieve stable imputation (56,57). For each imputed dataset, ten imputation cycles will be used. The imputation results will be validated by checking the distribution of imputed values against observed values. Rubin’s rule will be used to generate a complete single data set for analysis, encompassing the variation and uncertainty preserved in the multiple imputed data sets (55). Total costs, DAH90(HE), utility index score at day 90 and total QALYs with CCA, pre-imputed and imputed results will be presented for HFNT versus SOT comparison.” |
| 3. I see that parametric t-tests will be considered. Be aware that parametric tests must met quite strong assumptions to lead to reliable inference (e.g. Gaussianity, homoscedasticity, etc.). | We realise that parametric tests require conditions such as normality and homoscedasticity to be met. We plan to use alternative non-parametric methods such as the Mann-Whitney-Wilcoxon test and bootstrapped t-tests if these assumptions aren’t met. We have revised the manuscript to further clarify this approach.  The revised manuscript reflects these changes on Page 21, lines 347 to 350 and reads as follows:  “Significant differences at 5% level, using raw and imputed data, will be tested using two-sample t-tests of equality for normally distributed variables, and if assumptions of normality and homoscedasticity are not met, non-parametric Mann-Whitney-Wilcoxon test and bootstrapped t-tests will be used.” |
| 4. Regression models will be employed, but which models? Generalized linear models, generalized additive models, their mixed-effects counterparts? Survival models could also be employed. Please, provide more details, in particular of the way zero-inflation will be addressed (see e.g. Winkelmann, R. (2004). Health care reform and the number of doctor visits—an econometric analysis. Journal of Applied Econometrics, 19(4), 455-472; Alfò, M., & Maruotti, A. (2010). Two‐part regression models for longitudinal zero‐inflated count data. Canadian Journal of Statistics, 38(2), 197-216; Green, J. A. (2021). Too many zeros and/or highly skewed? A tutorial on modelling health behaviour as count data with Poisson and negative binomial regression. Health Psychology and Behavioral Medicine, 9(1), 436-455.) | There are a range of specific challenges to consider in the selection of the regression models. We started with a very general statement “Regression models will be fitted for mean costs and QALYS, with and without imputed values”, to highlight that we will follow good practice by considering the impact of imputation on regression results (which is uncommon). The issues we know we are likely to need to account for include potential baseline differences despite an RCT, missingness, potentially large numbers of zero values, long tails of very high cost patients, collinearity of baseline variables and correlation between costs and QALYs. As we don’t know the extent of these issues in the data until we examine it, we have pointed to the approaches we will consider (reflecting findings elsewhere that there is not necessarily one best model, Malehi et al 2015 Statistical models for the analysis of skewed healthcare cost data: a simulation study, Health Economics Review 5, 11). Later in the paragraph on regression approaches we have indicated that we will consider generalised linear or mixed models and test both log-link and more flexible forms. We have also indicated we will use seemingly unrelated regression.  Thank you for referring us to the additional references. We don’t think the first three papers represent the issue we are likely to need to model as those papers are based around an initial choice/selection of service. The intervention we are considering begins when the patient is unconscious in ITU – the ‘choice’ will just be to have joined the trial or not (which is not being evaluated) and from that point we start counting costs. Because every patient will stay at least one night in hospital, we think there will not be many zeros. Also, while we do have to check the potential for high number of zeros at the point of individual resource use, aggregating across resources for cost per patient further lowers the chance of zeros. While we need to look out for this latter issue, we think it is appropriate to downplay the potential importance of large numbers of zeros in this analysis (and have amended the text). The suggested papers did help us consider what ‘choice’ might exist later in the patient pathway e.g. after having left hospital and the ’choice’ to consume primary care services or not. If follow-up costs appeared to determine the difference between trial arms, a two-part model of some kind could be considered (another possibility could be a Heckman selection model, for example) for explaining follow-up costs. In practice however, we have found that differences between trial arms in hospital costs overshadow changes in costs in primary care. We think it is most likely that we will find this (we will be checking) because; one of the aims of the intervention is to lower length of hospital stay; the trial is not powered to detect differences in the consumption of primary care services, and we measure use of all primary care services so there is often more variation relative to hospital service use. This level of detailed analysis is also unfortunately not budgeted for but might make an interesting data set to explore in a future PhD or master’s dissertation.  The fourth suggested paper is likely to be more relevant because of the focus on highly skewed data, which could easily happen in both arms of the trial if some patients have a very long hospital stay. However, the focus on count data in this paper makes it of less relevance because our analysis focusses on costs aggregated across the patient pathway and is not therefore count data. However, we do think this is relevant to the analysis of the LOS variable itself and are passing this paper on to the trial statistician who is responsible for analysing that variable. We will consider the results of that analysis. Please note too, that we have also already indicated that skewness can be handled through the already indicated GLM.  The revised manuscript reflects these changes on Page 21 to 22, lines 359 to 361 that now reads as follows:  “We will examine data prior to selecting a regression model but will consider generalized linear and mixed models given their ability to handle skewness and large numbers of zeros, and will test log-link and more flexible forms to examine goodness of fit”. |
| **Reviewer 2 comments** |  |
| 1. This is a protocol paper describing the cost collection, valuation of resources, analysis methods and sensitivity analyses for an international trial for patients at high risk of postoperative pulmonary complications. The authors have submitted a well-written and highly detailed protocol paper. The planned analysis includes all the relevant components to a satisfactory economic evaluation utilizing cost-effectiveness and cost-utility analysis. However, there are some points I would like to raise: | We sincerely thank you for reviewing and providing your valuable feedback to improve this manuscript. We are pleased to know that you think the manuscript reads well and provides details of analyses.  Following your comments, we have further revised the manuscript, and these can be reviewed as track changes. |
| 2. Why choose to pool the results of the country-specific data? This will not inform any specific decision maker. It is better to use a base-case country for the main analysis and then conduct sensitivity analyses for the remaining countries. The results from the pooling analysis will not make sense to any decision maker and just seems rather non-important because the authors are already conducting the important country-specific analyses. | We agree that decision making at the country-level will be made using country specific analysis which is the planned base-case. However, we believe, pooling the results beyond using only country-specific data offers some benefits.  Country-specific trial pooled results allow us to use the larger sample of participants recruited within the structured framework of a rigorously conducted trial. This can help address uncertainty to an extent than using smaller country-specific samples.  Undertaking a pooled analysis can reveal patterns of resource utilization and outcomes across countries. Evidence of homogeneity can offer empiric evidence for generalizability across similar settings or conversely heterogeneity can highlight the important contextual factors influencing costs and outcomes across countries. Thus, we believe pooled analysis which will be presented following tests for heterogeneity can provide a valuable complement to the base-case country-specific analyses.  We have now added this to the discussion section in the revised manuscript on Page 28, Lines 456 to 457 which reads as follows:  “Moreover, the procedure of fully pooling the results can identify and provide empirical evidence of homogeneity or heterogeneity across these countries.” |
| 3. Why is the resource use for the family/friends not measured in time lost, as it is for the patient, and rather measured in time spent? The time spent with the patient is time lost at, for example, work? How do you value “time spent”? | Apologies for this confusion. The aim of the analysis is to account for productivity costs for patients and their families/friends. We have now revised the manuscript to uniformly report ‘time lost’ as the measure to account for these productivity costs.  The manuscript has been revised to reflect these changes on Lines 178, 227, 440 and Tables 1 and 2. |
| 4. I would like a more thorough description of the sub-group analysis – right now it seems forced and though out at the last minute. Why are these two sub groups chosen? Could there be other relevant sub groups that could be used in analysis? How about those that previously have had the same condition compared to the ones that are first-timers? How about social inequality aspects? We know that outcomes differ between patients with different socio-economic levels. | We agree that the suggested sub-groups could be relevant and could have differential cost-effectiveness implications. However, following discussion with the trial team and capacity at hand, we have restricted to the identification of sub-groups to those considered most relevant for the trial analysis.  We have now provided further rationale for the chosen subgroups in the revised manuscript.  The revised manuscript reflects these changes on page 26, Lines 419 to 424 that reads as follows:  “Heterogeneity will be assessed using sub-group analysis to improve robustness of our findings. Two potential sub-groups will be assessed: a) patients requiring escalation of oxygen therapy, as it may indicate treatment failure driving higher resource utilization and previous evidence suggests that HFNT may benefit patient subgroups receiving complete protective ventilation (68). b) patients with differential time on treatment (at least 8 or 24 hours) due to the potential impact on costs (for example, amount of oxygen used) and differential outcomes.” |
| 5. I would like a more comprehensive explanation of how the cost collection has been influenced by patients and the public. What does this mean, how was it done in detail, how did it differ between the countries and how does this affect the results? | The details of patient and public involvement with differences in data collection tools between countries in now reported in detail in supplementary table (Table S2, Appendix 1).  The revised manuscript on Page 8, lines 169 to 170 refers to the details in supplementary Table S2, Appendix 1. |
| 6. What is SOT? The first time this is mentioned in the Introduction it is mentioned using only the abbreviation and not explained beforehand. It is important to know what the comparison arm is and what is consists of. | Apologies for the confusion. SOT is standard oxygen therapy. The first time SOT is mentioned is in the Abstract (Page 2, line 30) and we have now spelled it out again in the introduction.  The explanation of what SOT entails is provided in the methods section on Page 7, Lines 143 to 147. We have reworded this paragraph to make this clearer.  The revised manuscript on Page 7, lines 143 to 147 reads as follows:  “Patients assigned to control arm (SOT) follow the exact same pathway except for a difference in the mode of oxygen therapy delivery following extubation. Patients receive oxygen via nasal prongs or non-rebreathing masks (not humidified and not heated) as is currently practiced. Patients receiving SOT are started on 30–40% inspired oxygen at a flow of 2 to 6 L/min, monitored and assessed as recommended (17).” |
